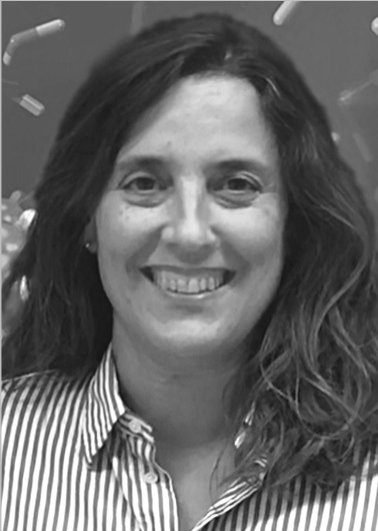# Molecular Photochemistry: Recent Developments in Theory

**DOI:** 10.1002/anie.201916381

**Published:** 2020-06-17

**Authors:** Sebastian Mai, Leticia González

**Affiliations:** ^1^ Photonics Institute Vienna University of Technology Gusshausstrasse 27–29 1040 Vienna Austria; ^2^ Institute of Theoretical Chemistry Faculty of Chemistry University of Vienna Währinger Strasse 17 1090 Vienna Austria

**Keywords:** excited states, molecular chemistry, non-adiabatic dynamics, photochemistry, quantum chemistry

## Abstract

Photochemistry is a fascinating branch of chemistry that is concerned with molecules and light. However, the importance of simulating light‐induced processes is reflected also in fields as diverse as biology, material science, and medicine. This Minireview highlights recent progress achieved in theoretical chemistry to calculate electronically excited states of molecules and simulate their photoinduced dynamics, with the aim of reaching experimental accuracy. We focus on emergent methods and give selected examples that illustrate the progress in recent years towards predicting complex electronic structures with strong correlation, calculations on large molecules, describing multichromophoric systems, and simulating non‐adiabatic molecular dynamics over long time scales, for molecules in the gas phase or in complex biological environments.

## Graphical abstract

**More light in chemistry**: This Minireview highlights the state‐of‐the‐art and emerging techniques in theoretical and computational photochemistry. This is a challenging and rapidly evolving field that relates time‐dependent spectroscopy with electronic structure theory and molecular dynamics for electronic excited states.

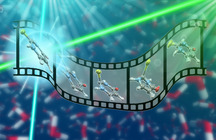

## 1 Introduction

Photochemistry is a broad and diverse branch of chemistry. A photochemical reaction is defined as a process that is triggered by the absorption of light and leads to an observable chemical change. When discussing photochemistry one also often considers photophysical processes where photon absorption and subsequent transitions change the electronic state of a molecule without necessarily leading to a chemical transformation. Furthermore, photochemistry is related to photobiology, which is concerned with biological processes involving light, such as in photosynthesis. The beauty of photochemistry lies in the fact that it is a complementary tool to thermally driven chemistry, enabling some reactions that are not possible thermally and occur very far away from chemical equilibrium. Driven by the desire to harvest these benefits, the last three decades have seen a renaissance of chemical reactions initiated by light with a wide range of applications,^1^ from synthesis to catalysis, from display technology to solar energy conversion, from analytical techniques to information technology, from biological imaging to optogenetics, and from diagnostics to therapy.

The allure of chemical reactions facilitated by light is that they are clean and often highly selective. Moreover, advancements in the field of laser‐based ultrafast spectroscopy in the last three decades have allowed researchers to understand and follow the temporal evolution of molecules after photoexcitation. Nonetheless, the discovery of excited‐state chemistry is often the result of serendipity, as reaction mechanisms involving electronic excited states are difficult to predict for two main reasons: On the one hand, many of the qualitative concepts and rules that predict thermally driven ground‐state reactivity are not applicable to electronically excited states.^2^ On the other hand, the details of decay processes of excited states governed by crossings of potential energy surfaces (PESs) most often elude experimental identification.^3^

Theoretical chemistry is particularly well‐suited to disentangle and predict reaction mechanisms at the molecular level, as it can locate relevant structures on the PESs and establish reaction paths connecting molecular structures. However, finding which of these critical features (structures and pathways) are relevant for a particular chemical reaction is not a trivial task. This endeavor becomes particularly difficult in situations where the molecular system exhibits many degrees of freedom and many electronic states, because this leads to a large number of critical features that need to be considered and a rapid growth in computational effort. To ease this search, multidimensional and multistate PESs can be explored with different excited‐state dynamics methods.

Here we present some of the computational challenges involved in mapping excited‐state PESs and simulating how the molecular system evolves according to these surfaces. Additionally, we provide a brief overview of popular and emergent methods, and showcase some of these methods with selected examples from our group and others.

## 2 Basics Concepts

**Figure 1:**
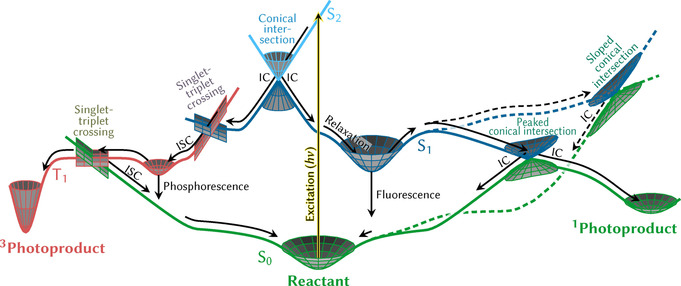
Schematic depiction of important photophysical and photochemical processes that might occur in a molecule after excitation by absorption of a photon (yellow arrow). After excitation to the *S*_2_ state, the system first encounters a conical intersection with the *S*_1_ state, where it undergoes internal conversion (IC) and can bifurcate towards the left or the right pathway. In the right branch, the system subsequently might relax to the *S*_1_ minimum, from where fluorescence can occur. Alternatively, the system could overcome a barrier to access conical intersections with the ground state, where two different types are shown that can be reached by following two different reaction coordinates (solid and dashed line). The peaked conical intersection allows for another bifurcation, which leads either back to the reactant or towards a singlet photoproduct. The sloped conical intersection is more difficult to access and usually favors only one decay pathway, here leading back to the reactant. In the left branch, the wave packet undergoes intersystem crossing (ISC) to the *T*_1_ state at a singlet–triplet crossing. There, the system can relax to the *T*_1_ minimum and eventually emit phosphorescence. Alternatively, it can escape the minimum and access a *T*_1_/*S*_0_ crossing to undergo intersystem crossing that leads back to the reactant or instead form a triplet photoproduct.

Mechanisms in photochemistry are mostly interpreted in terms of the different PESs that are accessed by a molecule after it is excited by light, before it returns to the electronic ground state and to either the initial or a different molecular geometry. Different PESs correspond to different electronic states, which typically change their electronic character and can cross in different ways, as depicted in Figure 1.

After excitation by absorption of a photon of energy *hν*, a nuclear wave function is promoted from the electronic and nuclear ground state to a bright excited state, thereby creating a wave packet that evolves in time. This temporal evolution arises because, in general, the forces the molecule is subjected to in the excited state are different from those in the ground state, or in simple terms, because the position of the ground‐ and excited‐state minima are not the same. This motion will direct the molecule towards a different geometry in the excited state. Depending on the topography of the excited‐state PES, the wave packet can relax to a new minimum, forming a long‐lived excited state that eventually decays back to the ground state through fluorescence, or continues visiting other regions of the PES. The wave packet might be able to overcome some potential energy barrier to reach crossing points, where so‐called non‐adiabatic population transfer to other surfaces is possible, that is, where the Born–Oppenheimer approximation is no longer valid and electronic and nuclear motion is coupled. This enables, among other things, a radiationless return to the reactant or the formation of a new molecule—a photochemical product. Internal conversion (IC) involves radiationless transitions between states of the same multiplicity (e.g. *S*_1_ and *S*_0_) and are mediated by crossing points that are called conical intersections, because of the double cone topography around the degeneracy point.^4^, ^5^ The discovery^6^, ^7^, ^8^ that IC is mediated by conical intersections and that such intersections are ubiquitous in chemistry and even biology^9^ could arguably be called one of the most significant breakthroughs in modern theoretical photochemistry. Crossing points between states of different multiplicity (e.g. *S*_1_ and *T*_1_) mediate intersystem crossing (ISC), provided spin–orbit coupling (SOC) is non‐negligible. Since SOC strongly depends on the electronic wave function of the two states and the nuclear charge of the atoms, ISC tends to be more common in molecules containing heavy atoms^10^, ^11^ than in purely organic molecules. However, ISC in molecules with only light atoms is also possible, as shown by early demonstrations from both experiment^12^, ^13^, ^14^, ^15^ and theory.^16^, ^17^, ^18^ ISC can provide a long‐lived state that can eventually undergo radiative (phosphorescence) or radiationless decay back to the ground state, or alternatively form some photochemical product. Note that in many systems, more than two multiplicities, additional states, or further processes might play a role.

**Figure 2:**
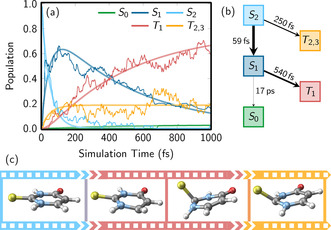
Non‐adiabatic dynamics simulations of 2‐thiouracil in the gas phase^19^ computed with the trajectory surface hopping method . a) Temporal evolution of the electronic state populations, b) corresponding kinetic model fit of the populations providing time scales and branching ratios among the pathways, c) a molecular movie with relevant geometries visited during the dynamics. Panels (a) and (b) are adapted from Ref. 19 with permission.

Knowledge about the PESs is key to understanding and predicting excited‐state chemistry.^2^, ^6^ However, in polyatomic molecules, PESs are high‐dimensional functions and thus complicated to characterize. Two general computational strategies have been developed to explore the PESs. In the *static approach*, one uses well‐established optimization techniques to identify potentially relevant important geometries (e.g. minima, transition states, crossing points), which can be connected to obtain relaxation pathways. The disadvantage of the static approach is that it might miss important geometries and thus a relaxation mechanism might be overlooked. Moreover, it does not predict time scales, cannot discern which pathways are the most important, and does not deliver quantum yields. A complementary strategy is the *dynamics approach*, in which the temporal evolution of the coupled nuclei and electrons is directly simulated. These so‐called non‐adiabatic molecular dynamics simulations are able to find directly the critical geometries that are actually visited by the system after excitation through a sort of molecular movie, as well as provide information about the relative importance of different decay channels and their time scales. One example is shown in Figure 2.

Despite the conceptual simplicity of the mentioned approaches, they are heavily hampered by the dimensionality and electronic complexity of the system. Both of these properties have a great impact on the cost of electronic structure calculations, which in the end are a key ingredient to both the static and the dynamics approaches. This implies that dynamics simulations typically cannot be carried out over long time scales (beyond a few picoseconds), thereby making slow or rare processes difficult to deal with. Hence, in practically all cases, one faces the problem of balancing computational cost with accuracy by considering different approximations.

## 3 The Challenges in Electronic Structure Theory

The static approach relies on quantum chemistry or electronic structure theory, that is, solving the electronic Schrödinger equation obtained from the Born–Oppenheimer (clamped‐nucleus) approximation. Besides electronic energies and geometrical structures, which allow characterization of the PESs of different electronic states, quantum chemistry can also provide coupling terms for non‐adiabatic transitions, for molecules in the gas phase, as well as embedded in different environments. There is a large array of electronic structure methods available; some can only deliver energies, most also provide gradients, and only a few can calculate couplings or Hessians, or are implemented in combination with environmental models. Thus, depending on the problem at hand, only one particular method might be suitable. It is not the aim of this Minireview to explain systematically the differences and capabilities of all available methods, as there is a good number of excellent books and reviews for this.^2^, ^20^, ^21^, ^22^, ^23^, ^24^, ^25^, ^26^ Instead, we would like to focus on several electronic structure methods that we perceive as popular or emerging, as well as related recent applications.

### 3.1 Multiconfigurational and Multireference Methods

At the top of the accuracy ladder of electronic structure methods is the full configuration interaction (FCI) approach, as it considers all possible electronic configurations in the wave function and, therefore, all electronic correlations. This is exact for a given basis set but unattainable except for the smallest systems and basis sets. Thus, correlated multiconfigurational methods^26^ are probably the next step on the ladder to calculate electronic excited states, as they can combine flexible wave functions with large amounts of static and dynamic electronic correlation, usually giving reliable energies whose accuracy is balanced all along the different geometries of the PESs—namely, from equilibrium to dissociation, including crossing points. Static correlation is important for ground‐state/excited‐state crossings, dissociation, untypical bonding situations, transition‐metal complexes with several oxidation states, many open‐shell states, and more. Dynamic correlation is relevant for obtaining accurate energies in general, even at equilibrium geometries. Methods such as CASSCF (complete active space self‐consistent field) complemented with CASPT2, NEVPT2, or MRPT2 (variants of second order perturbation theory on CASSCF), or MRCI (multireference CI) are established players in this field,^24^, ^26^, ^27^ but suffer from exponential scaling that limits the number of electrons and orbitals that can be considered in the so‐called active space. The active space is manually selected to contain the orbitals expected to be statically most correlated; with traditional CASSCF implementations, one is currently limited to less than 20 orbitals. Variations of CASSCF, such as RASSCF (restricted),^28^ GASSCF (generalized),^29^ SplitGAS,^30^ ORMAS (occupation‐restricted multiple),^31^ or LASSCF (localized)^32^ are emerging strategies in the quest to reduce this exponential scaling by defining different active subspaces. This allows a larger number of active orbitals, which in turn grants access to larger systems or higher accuracy.

The electronic states of transition‐metal complexes, especially those with partially filled d shells, provide particularly difficult challenges; this is one area where these new methods have demonstrated their value. Despite being small, Cr_2_ is one such challenging case due to the highly multiconfigurational character of its hextuple bond. In this case, 30 correlated orbitals in the SplitGAS partition scheme were shown to reproduce nicely the experimental dissociating potential.^33^ SplitGAS was also able to predict the energetic differences between the three different conformers of NiO_2_—a peroxo, superoxo, and a linear isomer—as well as several excitation energies of the copper anion [Cu(Cl)_4_]^2−^.^34^ However, that study^34^ also illustrated how challenging the correct choice of orbitals and spaces can be even for ground‐state problems. Thus, its routine applicability to excited states is still uncertain and needs further exploration. The LASSCF method, which exploits density matrix embedding fragmentation, is also a promising method to deal with strongly correlated systems, as demonstrated in dissociation^32^ and in the prediction of the low‐spin/high‐spin energy splitting of dinuclear iron complexes.^35^

One alluring way to approximate the FCI wave function with a polynomial, and thus more favorable, scaling is the density matrix renormalization group (DMRG) method.^36^, ^37^ Current calculations include up to 100 electrons in 100 orbitals. Although in really large molecules, this might not be enough, DMRG represents an overwhelming advance that enables benchmark calculations on strongly correlated systems with unforeseen accuracy. Applications of this method are flourishing, again with a particular focus on transition‐metal complexes, for example the Cr_2_ bonding problem,^37^ the Mn_4_CaO_5_ cluster model of the oxygen evolution complex of photosystem II,^38^ or problems concerning spin energetics.^37^, ^39^, ^40^, ^41^, ^42^, ^43^ Further studies on spin‐crossover systems have focused on the calculation of spin states in oxo‐Mn(Salen),^44^ Fe^II^porphin,^45^ dinuclear Fe^II^ compounds,^46^ chloro‐ligated iron(IV)‐oxo porphyrin,^47^ and metal monocarbonyl species.^48^ Early studies of excited states with DMRG were concerned with the dissociation curves of diatomic molecules,^49^, ^50^, ^51^ but genuine applications of photophysical and photochemical processes are slowly coming to light. Examples include the photochromic ring opening of spiropyran,^52^ the low‐energy spectrum of [2Fe‐2S] and [4Fe‐4S] clusters,^53^ the low‐lying singlet states of *trans*‐polyenes up to C_20_H_22_,^54^ the electronic structure of a naphthalene excimer,^55^ singlet donor–acceptor copolymers for singlet fission applications,^56^ photocyclizations,^57^ the delayed fluorescence in carbene‐metal amides,^58^ and excitations in carotenoids.^59^

**Figure 3:**
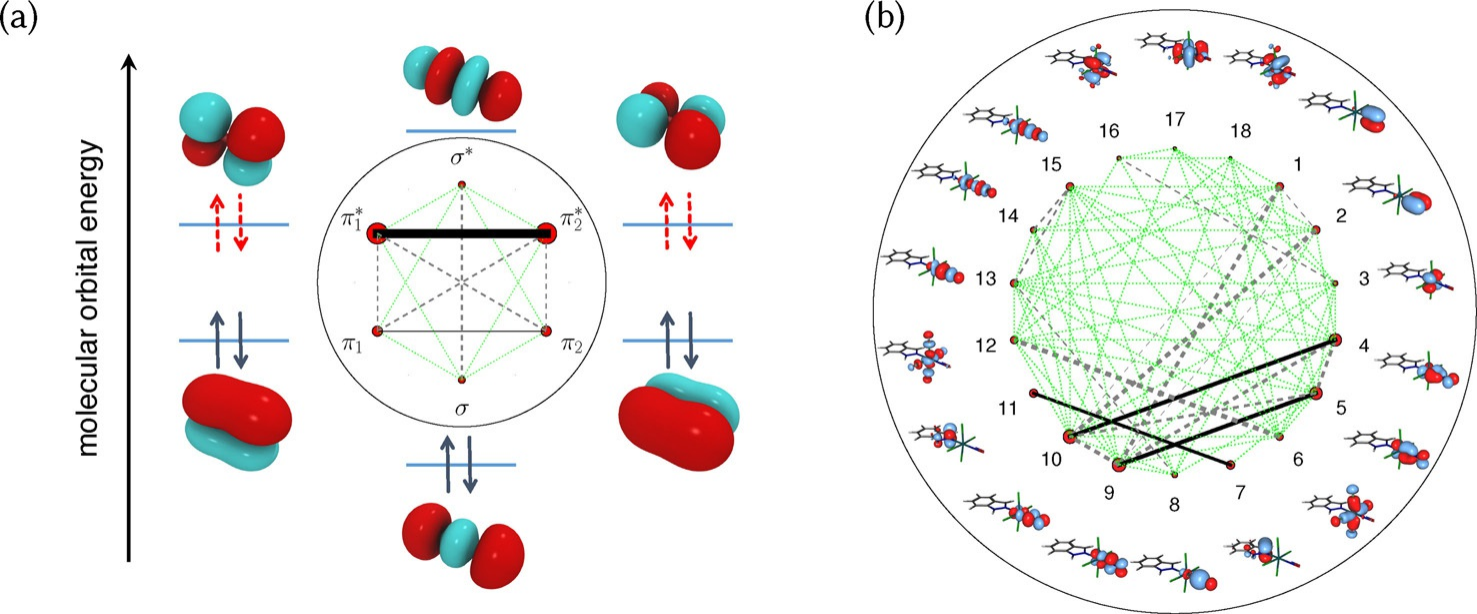
a) Molecular orbital diagram of the oxygen molecule in the ^1^Δ_*g*_ excited state,^64^ which consists of eight electrons in six orbitals and with the entanglement diagram in the center. Dashed arrows symbolize an electron pair that is equally likely to be in either of the two π* orbitals. The area of the red dot of each orbital is proportional to its single‐orbital entropy, whereas the thickness of the connecting lines is proportional to the value of the mutual information (see text for brief explanations). Reprinted from Ref. 64 with permission, Copyright (2017) Swiss Chemical Society. b) Entanglement diagram for *trans*‐[RuCl_4_(NO)(1*H*‐indazole)] with 18 orbitals.^68^ Reprinted from Ref. 68 with permission.

As DMRG allows inclusion of many orbitals, the question arises whether it is possible to devise an automated mechanism to identify the most important ones for a specific problem. The selection of the active space is usually done manually, which makes it tedious and possibly subjective. Although there are also other strategies to pick orbitals,^60^, ^61^ DMRG entanglement measures are very appealing, as exemplified in Figure 3. The diagrams present two important quantities: the single‐orbital entropy (red circles), a measure for how strongly the orbital occupation deviates from two or zero, and the mutual information (lines), which describes the static correlation of every pair of orbitals.^62^ These quantities indicate the orbitals that are most strongly statically correlated and should be part of the active space. Figure 3 a illustrates correlations for the oxygen molecule in the ^1^Δ_*g*_ excited state: as anticipated, the molecular orbitals generated from the 2p orbitals are all correlated, particularly the π* orbitals. In *trans*‐[RuCl_4_(NO)(1*H*‐indazole)] it is less clear which are the relevant orbitals without an entanglement plot (Figure 3 b). Based on these concepts, Stein et al. have proposed to iterate automatically these entanglement measures for active orbital space selection in a “black‐box” manner,^63^, ^64^, ^65^ with first applications reported for ferrocene^66^ and Ir complexes.^67^

Other routes besides DMRG exist for obtaining an accuracy approximately the same as FCI in excited‐state calculations.^69^, ^70^ For example, excited states can be computed with different variants of the Quantum Monte‐Carlo (QMC) stochastic approach,^71^, ^72^ with recent studies also presenting combinations of QMC with other techniques.^73^, ^74^ Applications of these techniques targeted, for example, azobenzene,^75^ solids,^76^ or retinal chromophore models.^77^ A very interesting variant of the QMC approach is the full‐CI Quantum Monte‐Carlo (FCIQMC) method,^78^, ^79^ where a determinant‐based FCI problem is not solved by storing all the coefficients simultaneously, but rather by “visiting” the most important determinants sequentially by a stochastic algorithm. Combined with CASSCF, FCIQMC has been used by Li Manni et. al.^80^ to obtain excitation energies of free‐base, Mg^II^, and Fe^II^ porphyrins using active spaces of 32 electrons and 29 orbitals at a modest computational expense. FCIQMC techniques have also been used to solve the intricate electronic structure of the singlet and triplet states of tetramethyleneethane.^81^

### 3.2 Single‐Reference Methods

Dynamical correlation alone can also be included using single‐reference methods. Here, Coupled‐Cluster (CC) methods and their variants,^82^ such as equation‐of‐motion CC (EOM‐CC) or approximate second‐order CC (CC2), the algebraic diagrammatic construction (ADC) scheme of the polarization propagator,^83^ as well as the much‐celebrated Time‐Dependent Density Functional Theory (TDDFT)^23^, ^84^, ^85^ are among the most widely used methods. In cases where static correlation does not play a role, such methods can achieve good accuracy in an almost “black‐box” fashion. Thus, it is not surprising that these—particularly TDDFT, but increasingly also ADC and CC—are the methods of choice to accompany many experimental investigations.^86^, ^87^, ^88^, ^89^ Promisingly, recent research has shown that ground state/excited state and excited state/excited state conical intersections can be described correctly by CC‐based methods.^90^, ^91^ We illustrate their broad applicability with a few examples, but plenty more exist. Grätzel and collaborators investigated the performance of such methods for the electronic absorption spectra of organic compounds employed in dye‐sensitized solar‐cell devices and found two density functionals to be particularly useful.^92^ Baerends et al. used TDDFT to provide a simple interpretation of the orbitals of Gouterman's four‐orbital model in Mg, Zn, and Ni complexes of porphyrin and porphyrazine.^93^ The ADC(2) method was employed to study the excitonic and charge‐transfer states of a bulk heterojunction model of a semiconductor^94^ and the photoisomerization mechanism of thiophenylazobenzene, an azoheteroarene photoswitch.^89^

Despite its undeniable success, TDDFT is well‐known to experience difficulties when dealing with charge‐transfer, double excitations, or Rydberg character. These problems can largely be alleviated by using specific exchange‐correlation functionals. Additionally, the Bethe–Salpeter equation in combination with many‐body Green's functions (GW/BSE) looms as an alternative to TDDFT with a similar computational cost.^95^ A good example of the superiority of GW/BSE over TDDFT is the calculation of excited states of cyanine dyes and polyenes.^95^ Another example of qualitatively and quantitatively good results obtained by GW/BSE can be found in the investigation of polymer‐fullerene model complexes for heterojunctions.^96^

### 3.3 Hybrid Methods Combining DFT and Wave Function Theory

Recently, there has been a lot of efforts to merge multireference wave function methods with DFT^97^ to better describe static correlation. Here we would like to highlight DFT/MRCI^98^ and the recently implemented multiconfigurational pair‐density functional theory (MC‐PDFT).^99^, ^100^ DFT/MRCI has been thoroughly tested and can represent ππ*, nπ*, Rydberg, and CT excitations and even doubly excited states in a balanced and reasonably accurate manner, in organic molecules as well as transition‐metal complexes without open‐shell ground states.

MC‐PDFT is less explored but it shows promising results. Recently it was used to evidence the different covalent and ionic contributions of the excited states of benzene^101^ and to predict singlet–triplet gaps in large polyacenes and polyacetylenes in combination with DMRG.^102^ A similar method is the state‐interaction state‐average spin‐restricted ensemble‐referenced Kohn–Sham (SI‐SA‐REKS) method,^103^ which combines dynamic correlation from DFT with a multiconfigurational approach. This method is already sufficiently developed to have analytical gradients implemented^104^ and has already been used in non‐adiabatic dynamics simulations, for example, of molecular rotors^105^ and biomimetic molecular switches.^106^

### 3.4 Linear Scaling Techniques, Multiscale Approaches, and Exciton Models

Although the capabilities of computers roughly doubles every two years, the exponential or polynomial scaling of the quantum chemical methods means that the size of molecular systems that can be treated nowadays does not suffice for many applications. This is why a number of other specific strategies exist for intrinsically large systems.

One endeavor is to apply low‐scaling techniques to the methods discussed above. Achieving linear scaling, where the computational cost scales linearly with the size of the system, is a very active field of research. Low‐scaling techniques have quickly evolved from calculating quantities in the electronic ground state to those in the excited state. Implementations, such as the Cholesky decomposition, resolution‐of‐the‐identity, and local approximations are now routinely implemented in methods such as CASSCF,^107^, ^108^ CC and ADC(2),^82^, ^109^ and TDDFT.^110^, ^111^ As a representative example, vertical excitation energies of tetrameric models of photosynthetic chlorophyll pigments (198–224 atoms) with ADC(2) and CC2 methods are no longer prohibitive.^112^ The last decade has also seen significant efforts to implement excited‐state electronic structure methods on graphics processing units (GPUs)—particularly by the Martínez group^113^, ^114^, ^115^—to achieve a significant speed‐up in, for example, TDDFT,^113^ CASSCF,^114^ or SI‐SA‐REKS.^115^ An unrelated recent implementation demonstrates sublinear scaling in TDDFT calculations of the excited states of large molecular systems, such as a pore of a covalent organic framework with almost 600 atoms.^116^

**Figure 4:**
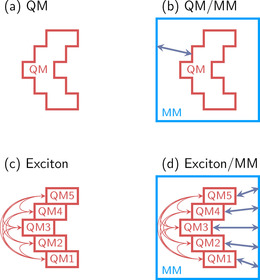
Schematic overview of different partition schemes to deal with large systems. a) A given QM system to be computed in the gas phase. b) A QM/MM partitioning, where a QM region and an MM region interact (arrow). c) A gas‐phase exciton model partition, where several QM chromophores are computed separately and interactions (arrows) are treated in a second step. d) A QM/MM partition, where an exciton model is used for the QM region and the solvent is treated by MM, including both QM‐MM and chromophore–chromophore interactions.

One strategy to compute large systems that are intractable with quantum chemistry alone is to divide the system into a small part that can be feasibly calculated with any of the quantum‐mechanical (QM) methods described and a larger part that is considered as the environment. The environment can be treated with a lower level of theory, usually with molecular mechanics (MM), as shown schematically in Figure 4 a,b; another cheaper QM method can also be used instead.^24^, ^97^, ^117^, ^118^ Such mixed approaches, also known as multiscale methods, were introduced in 1976 by Warshel and Levit^119^ as QM/MM to study the ground‐state properties of systems in biological environments. Nowadays this approach has been extended to include excited‐state properties in the QM region.^120^ QM/MM is now a vast field, with different implementations depending on the way the QM and MM parts are coupled or the number of QM layers considered.^121^ Different levels of theory are possible for the QM part. Two methods widely used in the QM part are TDDFT^122^, ^123^ and CASSCF, which are used to study chromophores in explicit solution or in the presence of biological environments (such as DNA, proteins, and lipids). Nowadays, applications treating the chromophore with ADC(2)^124^ or even CASPT2^125^, ^126^, ^127^ are increasingly common. For further computational efficiency, QM/MM can also be combined with the acceleration techniques mentioned above, for example GPU implementations of electronic structure methods.^128^ Another trend is the automatization of the laborious setup of QM/MM models in photobiological investigations, as shown, for example, in a specialized model for the family of rhodopsin proteins.^129^ For completeness, we note that it is also possible to calculate excited states in solution by combining QM with an implicit description of the solvent using a continuum model^130^ and this constitutes the easiest and by far most employed method to treat electronic states in solution and assist experiments.^131^

Exciton models are particular advantageous to describe multichromophoric systems. The idea is to divide the system into fragments, each of them containing only a single chromophore. The excitation energies and couplings of all fragments (Figure 4 c) are then combined to compute the system‐wide excitation energies by assuming certain approximations. Since the original definition of weakly interacting aggregates by Kasha,^132^ different variants of this strategy have been developed, depending on how the fragments are calculated and how the interactions between them are approximated.^133^, ^134^, ^135^ The level of theory employed to calculate each of the fragments could be any electronic structure method, but most of the current excitonic models are formulated within TDDFT, with a few attempts using wave function theory.^136^ Also here, the expense of the calculations can be alleviated with GPU acceleration.^113^ A favorite research area of exciton models is the study of electronic energy transfer in light‐harvesting processes occurring in photosynthetic pigment–protein complexes,^137^ but any system with at least two clearly defined and independent chromophores can be approximately calculated in this way. Exciton models can also be combined with multiscale approaches, for example, by embedding the independent chromophores in an environment described by MM^135^ (Figure 4 d).

## 4 The Challenges in Nuclear Dynamics Theory

**Figure 5:**
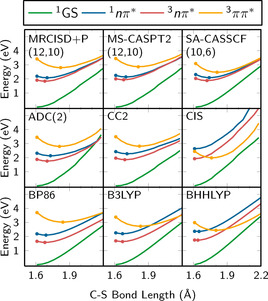
Potential energy curves of CH_2_S along the C−S bond calculated with different quantum chemical methods.^138^ Adapted from Ref. 138 with permission.

Besides the electronic structure challenge—which concerns the electronic problem in a fixed molecule—the other great challenge in computational photochemistry is the actual description of the nuclear motion induced by the forces exerted by the electrons. As the PESs will determine the dynamics of the system, it is indispensable that the chosen quantum chemical level of theory is able to describe the system adequately. Figure 5 shows potential energy curves for CH_2_S along the C−S bond length for a variety of single‐ and multireference methods. Although most methods qualitatively predict the same order of vertical excitations with acceptable accuracy, the stretching behavior of the C−S bond is very different, so that each of these methods predict different nuclear dynamics: specifically, while some will erroneously evidence intersystem crossing to the triplet states within 1 ps, others will not.^138^

Besides the challenges of quantum chemistry, the real problem is actually even more complex for all non‐adiabatic dynamics, namely, those involving more than one electronic state: how to describe the simultaneous, coupled movement of nuclei and electrons under their mutual attraction. As in Section 3, in the following we will not systematically list all available methods suitable for non‐adiabatic dynamics simulations, but we focus on a few selected examples of what we consider popular approaches and emerging techniques. For a more comprehensive overview on the topic, we refer the reader to several excellent recent reviews.^139^, ^140^, ^141^, ^142^

### 4.1 Methods Using Basis Functions

A principally exact description of nuclear motion including non‐adiabatic effects can be achieved by the explicit solution of the time‐dependent nuclear Schrödinger equation. To do so, one usually writes the nuclear wave function as a linear combination of some set of basis functions, combines this choice of basis functions with the nuclear Schrödinger equation to derive some set of equations of motion, and then numerically solves these. Hence, methods can be conveniently categorized by the type of basis functions they use. The simplest set of equations, and historically the first method for *quantum dynamics* (also known as the standard method),^143^, ^144^ employs a time‐independent, fixed basis called a grid. The disadvantage of this approach is that the number of grid points scales exponentially with the number of degrees of freedom, thus making it necessary to restrict the simulations to only a few (2–3) important coordinates. Despite this severe limitation, many important applications have made use of such quantum dynamics simulations, for example, in the areas of photobiology,^145^ optimal control of chemical reactions,^146^, ^147^ and laser‐induced electron dynamics.^148^

A significant step towards a wider applicability of quantum dynamics was the development of the multiconfigurational time‐dependent Hartree (MCTDH) method.^149^, ^150^ The underlying idea is to make the basis functions time‐dependent, namely, to use an algorithm to evolve the basis functions such that the wave function can be described with a basis as small as possible. In MCTDH, the algorithm is based on the variational principle. Compared to the standard quantum dynamics, the equations of motion become significantly more complex, but the more compact basis sets enable simulations with tens of degrees of freedom. Moreover, more specific variants of MCTDH, such as multilayered MCTDH,^151^ permit inclusion of about 100 degrees of freedom.^152^ The significantly expanded applicability of MCTDH is easily recognizable from its large number of diverse applications, such as thermally activated delayed fluorescence in organic light‐emitting diodes,^153^ organic photovoltaics,^154^ carbon monoxide photolysis in myoglobin,^155^ and correlated dynamics in highly excited molecules.^156^

A significant limitation of both standard quantum dynamics and MCTDH variants is that they require the full PESs to be computed prior to the dynamics simulations. For systems with many degrees of freedom, this becomes a major computational bottleneck, despite the advances shown in Section 3. Hence, much work was devoted to develop methods that do not require prior knowledge of the PESs. Instead, the goal was to carry out “on‐the‐fly” simulations, where the potential energies are only computed at the actually visited nuclear geometry. This can be achieved by employing localized basis functions, for example traveling multidimensional Gaussian functions. With this aim in mind, different methods were developed that differ in the equations that govern how the basis functions move. If the variational principle is employed for the latter purpose, then one obtains the Gaussian MCTDH (G‐MCTDH) method^157^ or the variational multiconfigurational Gaussians (vMCG) method.^158^, ^159^, ^160^ The latter is nowadays also available for on‐the‐fly simulations in the form of the direct‐dynamics vMCG (DD‐vMCG) method.^160^ Alternatively, the Gaussian basis functions can move according to classical mechanics, where they either follow forces averaged over multiple states—the basis of the multiconfigurational Ehrenfest (MCE)^161^ and ab initio multiple cloning (AIMC)^162^ methods—or follow single‐state forces but with new Gaussian functions created whenever needed, as in the ab initio multiple spawning (AIMS) method.^163^ All these methods employing Gaussian basis functions have the great advantage that they are inherently quantum dynamics methods that can formally produce the exact solution if the basis set is made large enough. Although this is not the case in real applications, all of these on‐the‐fly methods have proven to be highly useful for the study of excited‐state dynamics of small molecules, such as thymine,^164^ cyclobutene,^165^ pyrrol,^166^ and ethylene.^167^

### 4.2 Methods Using Trajectories

Another step down on the ladder of non‐adiabatic dynamics are those methods that employ point‐like, classical trajectories for the description of the nuclei. However, to allow for a description of non‐adiabatic effects, a quantum‐mechanical description of the electrons is retained, which is the reason these methods are termed “mixed quantum‐classical” methods. Two such methods were traditionally very popular. The classical Ehrenfest method^168^ employs classical trajectories that follow a force averaged over multiple states, for example after the electronic wave function developed into a linear combination of two electronic states during the traversal of a conical intersection. The second method is trajectory surface hopping (TSH),^169^ where trajectories always follow the gradient of one single electronic state, which is chosen stochastically based on how the electronic wave function evolves. A computational advantage of both methods is that each trajectory is independent from all the others, making the simulations easily parallelizable and thus widely used. Unfortunately, the independent‐trajectory approximation is also the largest source of error for these methods, as it precludes a correct description of nuclear quantum effects such as interference, tunneling, correct electronic decoherence,^170^, ^171^ or geometrical (Berry) phase effects.^172^, ^173^, ^174^ Hence, the last decade has seen a huge effort in developing more accurate and more versatile trajectory‐based non‐adiabatic dynamics methods, as documented in recent reviews.^141^, ^175^, ^176^, ^177^, ^178^, ^179^, ^180^ The popularity of TSH because of its simplicity and computational efficiency is recognized in the large number of applications, ranging from chemistry^181^, ^182^, ^183^, ^184^, ^185^ to biology,^186^, ^187^, ^188^, ^189^, ^190^ from surface chemistry^191^, ^192^, ^193^ to conjugated polyene materials,^194^ and from molecular rotors^116^, ^195^ to transition‐metal complexes.^11^, ^196^

Trajectory‐based non‐adiabatic dynamics has profited from the recent development of exact factorization theory,^197^ which is an alternative to the classical Born–Oppenheimer formalism for treating coupled nuclear–electron dynamics. The exact factorization formalism leads to a single (time‐dependent) PES governing the nuclear motion and, therefore, offers an elegant way to derive the exact force that classical nuclei should feel. Hence, exact factorization is a good starting point to derive more accurate trajectory‐based non‐adiabatic dynamics methods. The first such method—coupled‐trajectory mixed quantum‐classical (CT‐MQC) dynamics—was presented in 2014^198^, ^199^ and explicitly considers all trajectories at the same time, because the exact force involves a term depending on the distribution of nuclear positions. More recently, a decoupled version of CT‐MQC has been presented;^200^ it employs independent trajectories and is very much like TSH, hence it was christened decoherence‐induced surface hopping based on exact factorization (DISH‐XF). Both CT‐MQC and DISH‐XF have already been successfully applied in ring‐opening reactions of oxirane^199^ and cyclohexa‐1,3‐diene, respectively.^201^

### 4.3 Simulation of Observables

A very important aspect of any non‐adiabatic dynamics simulation should be its connection to the physical reality described by the experiment. Indeed, in many cases, non‐adiabatic dynamics simulations are carried out to directly model a particular experiment, most often from time‐resolved spectroscopy. The particularities of the experimental conditions are used to define the initial conditions and simulation setup. However, whereas an experiment returns a measured spectroscopic observable, the direct outcome of a dynamics simulation is composed of geometries, energies, and wave function coefficients, that is, quantities that are not directly comparable to the experimental data. Certainly, dynamics simulations are advantageous because the wave function and geometries can be used to directly understand the reaction mechanism, for example, how a particular bond or electronic state changes as a function of time over the course of the reaction. In other words, the simulation results can rather easily be re‐framed in language that chemists understand, for example, through a movie of molecular structures, electronic configurations, or energy barriers. In contrast, the interpretation of the spectroscopic data from a time‐resolved spectroscopic experiment into such chemical language is significantly more difficult. Clearly, a direct link between experiment and simulation can only be established if both return the same data.

**Figure 6:**
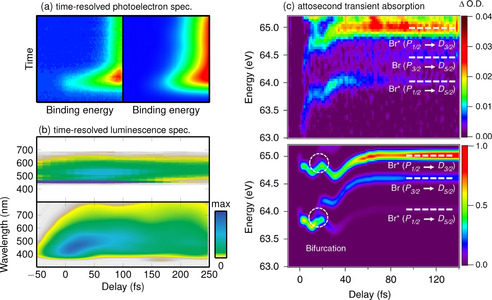
Examples of comparisons of experimental and simulated time‐resolved spectroscopic signals. a) Comparison of experimental and simulated time‐resolved photoelectron spectra of 2‐thiouracil. Adapted from Ref. 204 with permission. b) Time‐resolved luminescence spectrum of [Re(CO)_3_(imidazole)(phenanthroline)]^+^. Adapted from Ref. 11 with permission. c) Attosecond transient absorption spectrum of methyl bromide. Adapted from Ref. 207 with permission.

Therefore, a bridge between theory and experiment requires non‐adiabatic dynamics to go a step further and explicitly simulate spectroscopic observables, such as transient absorption spectra, photoelectron spectra, and others. Although most excited state dynamics simulations do not compute such observables, time‐resolved photoelectron spectra can be simulated within some approximations. Examples include acetone,^202^ uracil,^203^ 2‐thiouracil^204^ (Figure 6 a), or small metal atom clusters.^205^ Time‐resolved fluorescence spectra have been simulated for azobenzene,^206^ and time‐resolved luminescence spectra have been calculated for a rhenium transition‐metal complex^11^ (Figure 6 b). Transient absorption spectra were simulated in the past for systems such as rhodopsin^9^ and to complement attosecond experiments on methyl bromide^207^ (Figure 6 c). Whereas these spectra simulations are based on the computed energy differences and transition matrix elements, simulation of time‐resolved X‐ray scattering requires only to know the nuclear coordinate data. Examples have been presented for ethylene^167^ and metal‐organic Fe complexes.^208^

### 4.4 The Cost of Non‐adiabatic Dynamics and Possibilities To Alleviate It

The computational cost of non‐adiabatic nuclear dynamics simulations is the sum of a) the cost of simulating the motion of the nuclei (and the evolution of the electronic wave function) and b) the cost of the underlying electronic structure calculations used to obtain the PESs, gradients, and coupling terms. Both contributions depend very strongly on two parameters that influence the cost differently: the size of the system and the time scale of the simulation.

The dependence of the cost of electronic structure calculations on the system size is a well‐studied subject, and all the techniques presented in Section 3.4 aim to alleviate this expense. Such a cost reduction applies to both the precomputation of the PESs and on‐the‐fly ab initio calculations. In particular, GPU‐accelerated electronic structure methods for non‐adiabatic dynamics are already available.^114^, ^209^, ^210^

The size dependence of the nuclear dynamics method itself is a different matter. Full quantum dynamics methods using products of linear combinations of basis functions (i.e. standard quantum dynamics, MCTDH, G‐MCTDH) scale exponentially with the number of degrees of freedom. Hence, these methods are only affordable for small molecules or if only a subset of important degrees of freedom is included. Quantum dynamics methods that use a linear combination of multidimensional basis functions (e.g. vMCG, AIMS, AIMC, AIMCE) generally show a polynomial scaling and thus a better performance, although the actual scaling also depends on the employed equations of motion. Methods using classical nuclear trajectories are even more efficient, as these typically scale linearly with system size. This explains why most of the non‐adiabatic dynamics simulations of large heterogeneous systems—for example, molecules in bulk solution, nanomaterials, or biopolymers—are nowadays performed with a classical description of nuclear motion. However, new emerging techniques for non‐adiabatic dynamics, for example, based on ring‐polymer molecular dynamics,^211^, ^212^ might soon offer ways to include nuclear quantum effects with favorable scaling.

**Figure 7:**
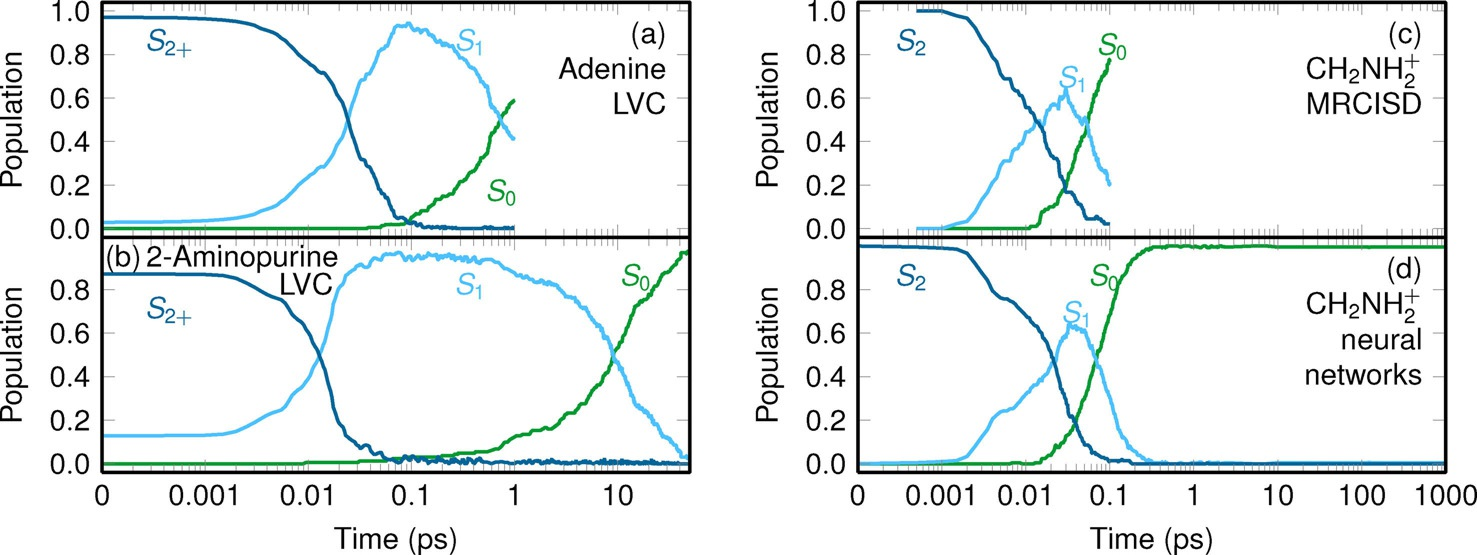
Examples of the temporal evolution of electronic population obtained from non‐adiabatic dynamics simulations over long time scales (note the logarithmic scale) using fitted potential energy surfaces. a,b) Comparison of the dynamics of adenine and 2‐aminopurine, two structural isomers with quite different photophysics:^220^ Whereas adenine deactivates to the ground state with a time constant of 1 ps, 2‐aminopurine takes about 13 ps. Both simulations were run with linear vibronic coupling potentials. Adapted from Ref. 213 with permission. c,d) Comparison of the dynamics of the methylenimmonium cation CH_2_NH_2_^+^.^221^ The expensive MRCISD method in (c) was used only to simulate 100 fs, whereas in (d) a neural network was used to fit the MRCISD PESs and enabled a total simulation time of 1 ns.^216^ Adapted from Ref. 216 with permission.

Although the computational cost of non‐adiabatic dynamics simulations scales “only” linearly with the simulated time, many non‐adiabatic processes extend over different time scales, ranging from femtoseconds—as in barrier‐free internal conversion in molecules—to nanoseconds, microseconds, or milliseconds—as in large‐scale environment‐response processes of proteins. To simulate processes that take significantly longer than the initial sub‐picosecond decay, it is necessary to substantially accelerate the non‐adiabatic dynamics, either by decreasing the computation time per step or by steering the dynamics towards the desired processes. One approach to decrease the computation time per step is to avoid the electronic structure calculations altogether; for example, by using fitted model potentials, vibronic coupling models,^213^ or machine learning potentials^214^, ^215^, ^216^ to describe the PESs. Recent examples of these techniques are shown in Figure 7. Figure 7 a,b show the results of non‐adiabatic dynamics simulations using linear vibronic coupling PESs.^213^ Figure 7 c,d compare the temporal evolution of the populations of the methylenimmonium cation CH_2_NH_2_^+^ obtained with MRCISD electronic structure calculations and neural network‐based PESs (about 120 times faster). Acceleration of slow or rare processes is nowadays feasible with steered non‐adiabatic dynamics, for example based on metadynamics,^217^ thus allowing relaxation pathways to be discovered that would otherwise take too long to reach with regular dynamics.^218^, ^219^

## 5 Conclusion

To summarize, theoretical methods for simulating photochemistry are booming and are able to deal with increasing complexity, be it the size of the molecule, electronic correlation, or quantum effects in nuclear dynamics. This Minireview provides a non‐exhaustive overview over new methods capable of calculating electronic excited states and of simulating their non‐adiabatic dynamics.

State‐of‐the‐art quantum chemistry can nowadays calculate vertical electronic energies of molecules with several hundreds of atoms, as long as no multiconfigurational situations are present, this means, mainly in organic closed‐shell systems with straightforward HOMO–LUMO transitions. The calculation of gradients for systems close to 1000 atoms is not yet routine, but it is expected that the use of GPUs and new acceleration techniques will make that possible very soon. For molecules with strong electronic correlation that need multiple electron configurations, methods such as DMRG seem very promising and a good number of recent applications have been highlighted in this Minireview, including spin states in multimetallic complexes, which are particularly difficult to deal with. In these cases, the size of the molecules to be treated does not exceed 100 atoms at best and the computational effort involved is still very large, so routine calculations are not yet possible. A large number of hybrid methods combining single and multireference approaches are emerging that exploit efficiency and accuracy; unfortunately, gradients and couplings are not available yet for many of them, thus making their use in dynamics less appealing. Systems that are too large to be described within one full quantum chemistry calculation, for example multichromophoric systems or chromophores within an environment, can be partitioned in different ways to treat the subsystems with different accuracy or by including approximated couplings between them.

Progress in non‐adiabatic dynamics has also been considerable. Aside from selecting a quantum chemical method that is able to describe correctly the PESs of the reaction where the nuclei will move, the choice of the nuclear dynamics method is very important. A sizable number of methods exist. The choice depends on how important the nuclear quantum effects are in the reaction at hand. From full quantum dynamics to mixed quantum‐classical methods, it is nowadays possible to calculate kinetics and other experimental observables to predict and interpret time‐resolved spectroscopic experiments with reasonable accuracy. The size and complexity of molecules that can be treated dynamically is strongly connected with the advances in electronic structure theory. However, the challenge to extend simulated time scales from a few picoseconds to nanoseconds or longer scales cannot be reliant only on the progress of quantum chemistry. The use of machine learning is receiving a lot of attention and the first successes have already been harvested, but a lot remains to be done.

The field of theoretical molecular photochemistry will continue to be an active and challenging playground. It is hoped that this Minireview will help to identify which method is best suited for which problem, to detail the current state‐of‐the‐art, and inspire future developments.

### Acknowledgement

We thank the University of Vienna and the Austrian Science Fund FWF (Grant I3987) for support, and L. Freitag for useful references.

## Conflict of interest

The authors declare no conflict of interest.

## Biographical Information

*Sebastian Mai obtained his degree in chemistry at the Friedrich Schiller University of Jena in 2012. In 2016, he received his PhD from the University of Vienna (with Leticia González). He was awarded the Loschmidt prize and Karl‐Schlögl prize for his dissertation. He continued as a postdoctoral researcher at the University of Vienna and since 2019 he has been at the Technical University of Vienna. His research focuses on excited‐state electronic structure theory, photophysics, and photochemistry, with a particular emphasis on developing the non‐adiabatic molecular dynamics software package SHARC*.

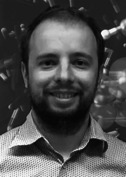

## Biographical Information

*Leticia González obtained her PhD in 1998 (with Otilia Mó and Manuel Yáñez) at the Universidad Autónoma de Madrid. After postdoctoral research with Jörn Manz at the Freie Universität Berlin, she habilitated in 2004. From 2007 to 2011, she was Professor at the Friedrich Schiller University of Jena and since 2011 Full Professor at the University of Vienna. Her research focuses on chemistry of excited states, combining quantum chemistry calculations, and developing non‐adiabatic dynamics methods. She was awarded the Dirac Medal in 2011 and the Prize for Excellent Research from the RSEQ in 2019*.